# Characterization of Monoclonal Antibody Glycan Heterogeneity Using Hydrophilic Interaction Liquid Chromatography-Mass Spectrometry

**DOI:** 10.3389/fbioe.2021.805788

**Published:** 2022-01-11

**Authors:** Sumit K. Singh, Kelvin H. Lee

**Affiliations:** ^1^ Department of Chemical and Biomolecular Engineering, University of Delaware, Newark, DE, United States; ^2^ School of Biochemical Engineering, Indian Institute of Technology (BHU) Varanasi, Varanasi, India

**Keywords:** glycosylation, glycan, monoclonal antibody, CHO cell, hydrophilic interaction liquid chromatography

## Abstract

Glycosylation is a critical quality attribute of monoclonal antibody (mAb) therapeutics. Hydrophilic interaction liquid chromatography-mass spectrometry (HILIC-MS) is an invaluable technology for the characterization of protein glycosylation. HILIC/MS-based glycan analysis relies on the library search using Glucose Units (GU) and accurate mass (AM) as the primary search parameters for identification. However, GU-based identifications are gradient-dependent and are not suitable for applications where separation gradients need to be optimized to analyze complex samples or achieve higher throughput. Additionally, the workflow requires calibration curves (using dextran ladder) to be generated for each analysis campaign, which in turn, are used to derive the GU values of the separated glycan species. To overcome this limitation, we employed a two-step strategy for targeted glycan analysis of a mAb expressed in Chinese Hamster Ovary (CHO) cells. The first step is to create a custom library of the glycans of interest independent of GU values (thereby eliminating the need for a calibration curve) and instead uses AM and retention time (RT) as the primary search variables. The second step is to perform targeted glycan screening using the custom-built library. The developed workflow was applied for targeted glycan analysis of a mAb expressed in CHO for 1) cell line selection 2) characterizing the day-wise glycan evolution in a model mAb during a fed-batch culture, 3) assessing the impact of different media conditions on glycosylation, and 4) evaluating the impact of two different process conditions on glycosylation changes in a model mAb grown in a bioreactor. Taken together, the data presented in this study provides insights into the sources of glycan heterogeneity in a model mAb that are seen during its commercial manufacturing.

## 1 Introduction

Monoclonal antibodies (mAbs) are the fastest-growing modality in the biopharmaceutical industry with application towards treating many diseases including cancer (Grilo and Mantalaris, 2019). An important feature of mAbs that is crucial for safety and efficacy is glycosylation (Eon-Duval et al., 2012). About two-thirds of all the approved biologics include glycosylation as a key post-translational modification bears testimony to its importance in the development of biologics (Walsh, 2018).

The most common type of glycosylation observed in mAbs is N-glycosylation. In this type of glycosylation, glycans are attached to the nitrogen atom of an Asparagine (Asn) residue with a consensus sequence of Asn-X-Ser/Thr, where X is any amino acid except proline (Schwarz and Aebi, 2011). Moreover, N-glycans also possess a common five-membered trimannosyl chitobiose core, to which N-acetylglucosamine, galactose, and sialic acid get added in a probabilistic fashion, resulting in considerable observed glycan complexity and heterogeneity (Fisher et al., 2019). The overwhelming diversity of possible glycan types on a mAb necessitates analytical methods that can characterize the mAb glycosylation at different levels of protein architecture (Jensen et al., 2012; Reusch et al., 2015a; Reusch et al., 2015b). These include analysis at the level of intact proteins, protein subunits, peptides, and released glycans often utilizing liquid chromatography with mass spectrometry and/or fluorescence detection (Kaur, 2021). In addition, other approaches include capillary electrophoresis-mass spectrometry (CE-MS), capillary electrophoresis-laser induced fluorescence detection (LIF), high-performance anion-exchange chromatography with pulsed amperometric detection (HPAEC-PAD), and/or nuclear magnetic resonance (NMR) spectroscopy (Duivelshof et al., 2019).

Among the available analytical methods for glycosylation analysis, a common approach is to release the glycans from the protein by hydrolyzing the side-chain amide group of the asparagine residue of the protein by treating the analyte with Peptide-N-glycosidase F (PNGase F) enzyme (Hilliard et al., 2017). The released glycans are either permethylated, reduced, or labeled with a fluorophore dye before analysis with LC-MS to enhance the optical detection and MS ionization efficiency (Jensen et al., 2012). Chromatographic analysis of glycans is typically performed with hydrophilic-interaction chromatography (HILIC) because of the relatively lower back pressure (due to high fraction of organic solvents during separation) making the analysis amenable to the use of ultra-high-performance liquid chromatographic columns (Lauber et al., 2015). This fact, in turn, facilitates improved resolution under the separation conditions.

HILIC-MS analysis involves the identification of each of the glycans by conversion of the retention time to Glucose Units (GU) (Domann et al., 2007). The GU values of each of the glycans is a specific property of the fluorescent tag used for labeling, and therefore each tag will have a unique GU value for a particular glycan. The most widely used commercial fluorescent tag that is currently used for released glycan analysis is RapiFlour-MS (RFMS), because its properties enable rapid labeling, high sensitivity FLR measurements, and improved MS ionization in the positive mode of operation (Hilliard et al., 2017). Other commonly used fluorescent tags include 2-aminobenzamide (2-AB), 2-aminoanthranilic acid (2-AA), and 4-aminobenzoic acid 2-(diethylamino)ethyl ester (Zhou et al., 2017). While the released glycan assays using GU-based identification followed by confirmation using mass spectrometry have certainly improved analytical performance, this workflow is gradient-dependent (works only with the gradient used for generating the search library). As such, the GU-based glycan workflows are not well-suited for applications where separation gradients need to be optimized to analyze complex samples or achieve higher throughput.

In this work, we developed an alternative workflow that overcomes this limitation by using accurate mass (AM) as the primary search parameter. For this, the search library (from Waters Corp) was modified by removing the GU-information and incorporating the AM data for all the glycan candidates in the search library. Next, the retention time data were included for the glycans of interest as a confirmatory screening variable. Finally, the workflow was applied for screens of the following: 1) cell line selection 2) characterizing the day-wise glycan evolution in a model mAb during a fed-batch culture, 3) assessing the impact of different media conditions on glycosylation, and 4) evaluating the impact of two different process conditions on glycosylation changes in a model mAb grown in a bioreactor.

## 2 Materials and Methods

### 2.1 Monoclonal Antibody Fed-Batch Cell Culture

Recombinant CHO-K1 Clone A11 expressing the anti-HIV antibody VRC01 (IgG1) was obtained from the Vaccine Research Center at the National Institutes of Health (NIH). Working cell bank cells (1 ml: 90% media and 10% DMSO frozen in liquid nitrogen) were thawed directly into 125 ml vented shake flasks with 30 ml working volume containing ActiPro medium (GE Healthcare) with 6 mM L-glutamine (Sigma-Aldrich) (media 1) and a parallel bank was thawed using a proprietary chemically-defined Medium 2, and a proprietary chemically-defined Medium 3. Cultures were maintained in a 5% CO_2_ incubator at 37°C in a 1-inch orbital shaker at 135 rpm. Cells were passaged five times, in the exponential growth phase (every 2–3 days) to a target cell density of 0.4 × 10^6^ cells/ml, before inoculation into the fed-batch.

Fed-batch was carried out in shake flasks and ambr250 bioreactors (Sartorius) using the appropriate feeds for each medium and cells harvested at day 14 or 70% viability whichever came first.

### 2.2 Protein-A Purification

The mAb from the different cell lines were purified from the cell culture harvest fluid using a 5 ml HiTrap Protein-A HP column (Cat. 17040201, GE Healthcare, Uppsala, Sweden). Briefly, protein A column was equilibrated with 50 mM phosphate containing 150 mM NaCl, pH 7.5 (5CV). Next, the cell culture harvest was loaded onto the column such that a residence time of 4 min was obtained. The column was subsequently washed with the equilibration buffer (2CV). Elution was performed using 100 mM glycine buffer, pH 3 (1.5CV). Before subsequent loading, the column was reconditioned using washing with 2 M NaCl (2CV) and 0.5 M NaOH (2CV). The concentration of the elute was determined using UV-Spectrophotometer (Cat. ND-2000, Thermo Fisher) by recording the absorbance at 280 nm. The samples were buffer exchanged into water +0.1% formic acid before mass spectrometric analysis.

### 2.3 Glycan Analysis With Library Searching

#### 2.3.1 Glycan Release

Briefly, the purified samples were concentrated to 2 mg/ml in water +0.1% formic acid. A 7.5 ml aliquot (15 mg) was diluted with 15.3 ml of water. To this, a 6 ml of a 5% RapiGest solution (Cat. 186002123, Waters) was added. The solution was heated at 95°C for 5 min and then cooled to the ambient temperature. Next, a 1.2 ml aliquot of Rapid PNGase F was added and mixed by aspiration. The sample solution was incubated at 55°C for 5 min and cooled at room temperature for 5 min.

#### 2.3.2 Glycan Labeling

Following the deglycosylation from the mAb, the released glycans were labeled with RapiFluor-MS™ (RFMS). A 24-reaction kit was used and the RFMS reagent (23 mg) was dissolved in 131 ml of anhydrous DMF. A 12 ml aliquot of this solution was added to the deglycosylation mixture and mixed by aspiration. The labeling reaction was allowed to proceed at room temperature for 5 min. The reaction was then quenched with the diluting reaction mixture with a 360 µl aliquot of acetonitrile (ACN).

#### 2.3.3 Glycan Purification

The labeled glycans were purified using a HILIC µ-elution plate. The wells of the µ-elution plate were conditioned with 200 µl aliquots of water (three times). The steps in purifying the labeled glycans from other byproducts involve the following steps: equilibration with 200 µl of 85% ACN, sample loading (∼400 µl), wash with 1% formic acid, 90% ACN, and finally elution with 30 µl SPE Elution Buffer (200 mM ammonium acetate in 5% ACN). The eluate was diluted with 310 µl of GlycoWorks SPE diluent (DMF/ACN) and mixed by aspiration.

#### 2.3.4 UPLC-MS

The purified glycans from the above step were separated on a Waters ACQUITY UPLC system^®^ using Waters ACQUITY UPLC Glycan BEH Amide column (2.1 × 150 mm, 1.7 mm particle size, 130 Å pore size). A 50 mM ammonium formate solution (pH = 4.4) was used as mobile phase A and a 100% ACN solution was used as mobile phase B. Other parameters for chromatographic separation of glycans included column temperature: 60°C and 75–54% gradient of mobile phase B over 35 min at a flow rate of 0.4 ml/min. The separated glycans were detected using a fluorescence (FLR) detector with an excitation wavelength of 265 nm and an emission wavelength of 425 nm. MS data were recorded on a Bioaccord^®^ Waters MS system operated in positive ion mode. MS parameters included: m/z range: 50–2000, capillary voltage: 1.5 kV, cone voltage: 45 V. The MS was calibrated using ACQUITY RDa calibrant and wash solution (Cat. 186009012, Waters) and lock mass calibration was performed using ACQUITY RDa lock mass solution (Cat. No. 186009012, Waters).

#### 2.3.5 Data Analysis

The glycan peak annotation was performed using the Glycan Assay (FLR with MS confirmation) workflow within UNIFI. Initially, a calibration curve is constructed by running a dextran ladder labeled with RFMS (Cat No. 186007982, Waters) using the above-described method conditions. The components of the dextran ladder, G4 through G12 with the expected retention times are noted. Next, the retention times of the released glycans are converted into GU values using the calibration curve. The GU values of the assigned species are then matched with the potential glycan structures in the RFMS glycan GU scientific library in UNIFI with a GU tolerance of 0.2 units and mass error of 15 ppm. The relative abundance of any mass confirmed glycan species was determined from the dividing FLR peak area of that species with the total summed peak area of all the identified glycans. Statistical analysis for comparing mAb glycosylation from different cell lines was done by two-way ANOVA using Tukey post-test with Graphpad software version 8.4.

## 3 Results

### 3.1 Glycan Analysis of VRC01 mAb Using the Standard Method

**FIGURE 1:**
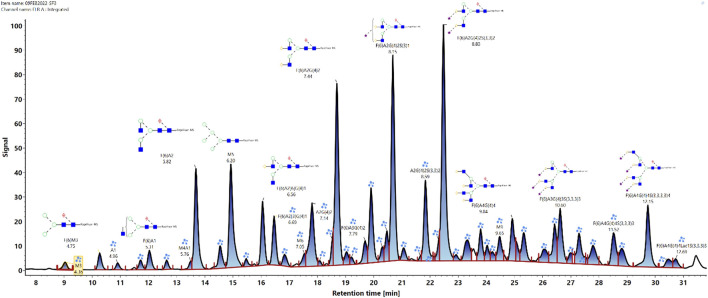
Released N-glycan map from the FLR channel of the HILIC- UPLC-FLR-MS analysis of VRC01 mAb. In total 53 glycans were identified and annotated (44 were identified and annotated based on GU and accurate mass, while 9 of them were based only on GU values).

**FIGURE 2:**
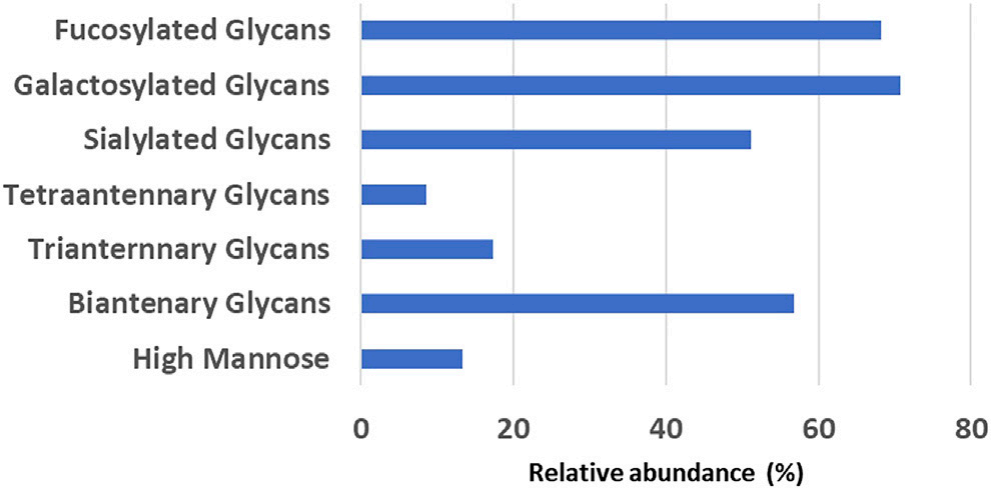
The relative abundance levels of various glycan attributes obtained by summing the corresponding normalized abundance levels for each glycan species that possess that attribute (listed in Table 1).

The glycan analysis of the VRC01 using the standard library search method resulted in the identification of 53 confirmed N-glycans, out of which 44 were assigned based on both GU values and accurate mass, while the remaining nine glycan species were annotated based on GU values alone (Figure 1). These nine glycan species were low abundance glycans (<2%) and co-eluted with a more abundant glycan species that potentially resulted in suppression of its mass spectrum. Table 1 lists out the identified glycans with their corresponding normalized abundances expressed as the total area of the FLR peak. The relative abundances of these glycans spanned in the range from 0.1% (A2[3]G(4)1S(6)1**)** to 11.92% (F(6)A2G(4)2S(3,3)2). A total of 18 glycans were base-line resolved, while 35 glycans co-eluted with a more abundant species but were identified and mass-confirmed with this method. Out of the 53 identified glycans, 25 were complex biantennary, 12 were complex triantennary, five were complex tetra antennary, five were high mannose, and six were hybrid glycans. The individual glycan species identified for the VRC01 mAb were grouped into various glycan attributes and are shown in Figure 2. The percentage calculated is the sum of individual normalized amounts (Table 1) for each glycan species contributing to the overall abundance of a given attribute. As it is evident from Figure 2, the majority of glycans are complex biantennary glycans (∼57%). High mannose forms also constitute ∼13.2% of the overall glycans of VRC01 indicating the presence of immature glycoforms. Of the total identified VRC01 glycans, ∼70% of the glycans were galactosylated, ∼51% of them were terminally sialylated, and ∼68% were core-fucosylated. The higher levels of sialylation observed in VRC01 is an unusual feature generally not observed with mAbs, thereby suggesting it to be an important quality attribute of VRC01. The heavy sialylation level seen in the VRC01 was attributed to glycans occupying the light chain that comprised of heterogenous population of bi-, tri-, and tetraantennary, core fucosylated complex galactosylated and terminally sialylated glycans.

**TABLE 1:** List of the confirmed N-glycans derived from VRC01 mAb analyzed in the study. Also included are the observed retention times, normalized abundance (% amount), expected and observed GU values, expected and observed masses, the predominant charge state, and the response units. The highlighted glycan species are those that are shortlisted to be monitored using the screening workflow.

SL. No	Component name	Observed RT (min)	Glycan units	Observed mass (Da)	% Amount (%)	Expected glycan units	Expected mass (Da)	Charge	Response
1	M3	9.04	4.3583	1,221.507	0.43	4.3633	1,221.502	2	31886133
2	F(6)M3	10.27	4.752	1,367.564	0.81	4.7617	1,367.56	2	60887988
3	A1	10.9	4.9581	1,424.587	0.33	4.9699	1,424.582	2	24741016
4	M4	11.72	5.2148	1,383.558	0.46	5.3077	1,383.555	2	34666288
5	F(6)A1	12.03	5.3126	1,570.643	1	5.2906	1,570.64	2	75026269
6	A2	12.64	5.5003	1,627.665	0.42	5.4619	1,627.661	2	31299243
7	M4A1	13.5	5.7619	1,586.637	0.32	5.7402	1,586.635	2	24184490
8	F(6)A2	13.68	5.8169	1,773.727	4.98	5.7879	1,773.719	2	3.74E+08
9	F(6)A1[3]G(4)1	14.54	6.0808	1,732.698	1.24	6.095	1,732.692	2	93050430
10	M5	14.92	6.1996	1,545.611	5.75	6.1692	1,545.608	2	4.31E+08
11	A2[3]G(4)1	15.47	6.3716	1,789.718	0.36	6.29	1,789.714	2	27222853
12	F(6)A2[6]G(4)1	16.05	6.559	1,935.777	2.87	6.5337	1,935.772	2	2.15E+08
13	F(6)A2[3]G(4)1	16.46	6.6921	1,935.779	2.3	6.6633	1,935.772	2	1.72E+08
14	F(6)A2[6]BG(4)1	16.83	6.8134	2,138.856	0.66	6.8	2,138.851	2	49258887
15	M6	17.54	7.0486	1,707.662	0.61	7.1138	1,707.661	2	45834299
16	A2G(4)2	17.81	7.1397	1,951.772	4.05	7.0955	1,951.767	2	3.04E+08
17	M6 D1	18.07	7.2303	854.8377	0.21	7.14	821.7959	2	15499336
18	A2BG(4)2	18.52	7.3836	2,154.865	0.78	7.35	2,154.846	3	58458127
19	F(6)A2G(4)2	18.69	7.4413	2,097.832	8.91	7.4266	2,097.825	2	6.68E+08
20	M5A1G(4)1	19.03	7.5596	1,910.739	0.69	7.431	1,910.74	2	51800792
21	F(6)A2BG(4)2	19.21	7.6232	2,300.913	0.31	7.605	2,300.904	3	23189435
22	F(6)A3G(4)2	19.69	7.7936	2,300.912	1.15	7.7585	2,300.904	3	86301406
23	F(6)A2[6]G(4)1S(6)1	19.9	7.8696	2,226.876	3.7	7.84	2,226.867	2	2.77E+08
24	M7 D1	20.3	8.0131	1,869.719	0.65	8.0285	1,869.714	2	48971172
25	A2G(4)2S(3)1	20.47	8.0732	748.6280	1.29	8.06	729.282	2	96888102
26	F(6)A2G(4)2S(3)1	20.68	8.1502	2,388.927	9.36	8.124	2,388.92	2	7.01E+08
27	F(6)A2[3]G(4)1Sg(6)1	21.06	8.2939	2,242.868	0.65	8.3057	2,242.862	2	48619741
28	F(6)A2G(4)2S(6)1	21.65	8.5146	1,195.4673	0.14	8.55	1,394.5757	2	10806796
29	A2G(4)2S(3,3)2	21.83	8.5856	2,533.966	3.97	8.57	2,533.958	3	2.97E+08
30	A2[3]G(4)1S(6)1	22.11	8.6935	694.6104	0.1	8.69	586.5778	2	7558824
31	F(6)A2BG(4)2S(6)1	22.3	8.7666	2,592.009	0.67	8.82	2,591.999	3	50024309
32	F(6)A2G(4)2S(3,3)2	22.47	8.8339	2,680.025	11.92	8.85	2,680.016	3	8.94E+08
33	A2G(4)2S(3,6)2	22.92	9.0107	2,533.965	0.33	9	2,533.958	3	24788235
34	A3G(4)3S(3)1	23.33	9.1737	2,608.012	1.57	9.23	2,607.994	3	1.18E+08
35	A2G(4)2S(6,6)2	23.8	9.3668	845.6598	1.48	9.415	869.3230	2	1.11E+08
36	F(6)A3G(4)3S(3)1	24.02	9.4614	2,754.06	0.68	9.538	2,754.052	2	50906409
37	F(6)A2G(4)2S(6,6)2	24.21	9.5405	2,680.015	0.47	9.69	2,680.016	2	35023310
38	M9	24.47	9.6488	2,193.825	1.32	9.6667	2,193.819	2	98775879
39	F(6)A4G(4)4	24.92	9.8416	2,828.098	2.12	9.915	2,828.089	3	1.59E+08
40	F(6)A3Lac3	25.05	9.8994	1,537.1048	0.68	9.91	1,529.1198	2	50960194
41	F(6)A3G(4)3Lac1	25.33	10.019	2,828.1	1.72	10.015	2,828.089	3	1.29E+08
42	A3S(6)1G(4,4,3)3S(3,6)2	26.04	10.3369	3,190.18	1.1	10.31	3,190.185	3	82361071
43	M10 a3D1,a3D3,a2D4(2)	26.42	10.5103	2,355.882	2	10.53	2,355.872	2	1.5E+08
44	F(6)A3G(4)3S(3,3,3)3	26.62	10.6002	3,336.262	2.97	10.6267	3,336.243	3	2.23E+08
45	A3G(4)3S(3,3,6)3	26.99	10.774	2,879.0101	0.51	10.72	2,862.6845	3	38209650
46	F(6)A3G(4)2Ga(3)2Sg(6)1	27.29	10.9168	978.3740	1.99	10.94	940.687	2	1.49E+08
47	F(6)A4G(4)4Lac1	27.53	11.0304	3,193.235	0.14	11.01	3,193.221	3	10417180
48	F(6)A3G(4)3Lac2	27.79	11.1559	1,065.41	1.52	11.16	1,059.7256	2	1.14E+08
49	F(6)A4G(4)4S(3,3,3)3	28.52	11.5199	3,701.393	2.02	11.52	3,701.375	3	1.51E+08
50	F(6)A3G(4)3Lac1S(3,3,3)3	28.81	11.666	3,701.389	1.39	11.71	3,701.375	3	1.04E+08
51	F(6)A4G(4)4S(3,3,3,3)4	29.73	12.148	3,992.491	3.7	12.17	3,992.471	3	2.77E+08
52	F(6)A3G(4)3Lac2S(3,3,3)3	30.47	12.5441	4,066.515	0.66	12.52	4,066.508	3	49654386
53	F(6)A4G(4)4Lac1S(3,3,3)3	30.73	12.6875	4,066.512	0.54	12.7	4,066.508	3	40618466

Abbreviations: F, fucose; G, galactose; A1, monoantennary; A2, biantennary; Sg, N-glycolylneuraminic acid; Ga, a1,3-linked galactose.

Based on the VRC01 glycan profile using the standard analysis method, 12 glycan species were selected for the monitoring using the glycan screening method (highlighted in Table 1). The selection of these species was made based on the relative % amount, with each of these glycan species present in an amount greater than or equal to 3%. Cumulatively, these 12 glycan species constitute ∼65% of overall VRC01 glycans.

### 3.2 Glycan Screening Method

The first step in the development of the glycan screening method was to prepare a library of glycan species devoid of GU value information. This step was accomplished by deleting the corresponding GU values of each of the glycan species from the existing waters-commercial glycan library. The Waters commercial glycan library contains GU values of 168 N-linked glycans derived from trastuzumab, infliximab, etanercept, NISTmAb, human serum polyclonal IgG, mouse serum polyclonal IgG, erythropoietin, bovine fetuin, and yeast invertase (Hilliard et al., 2017). The rationale of choosing these glycoproteins for the generation of glycan search library was two-fold: 1) to comprehensively cover the three classes of N-linked glycans namely, high mannose, complex, and hybrid structures; and 2) to provide the extensive coverage of the N-linked glycans that are routinely attached to the therapeutic mAbs and other biopharmaceutical products. Each of these glycans in the commercial library contains information about the corresponding GU values and accurate mass information. The glycan screening library, therefore, is composed of these 168 N-linked glycan species with their only corresponding accurate mass information (Supplementary Table S1). Further, the retention time information of the VRC01 glycan species that are shortlisted for screening was updated to the glycan screening method from the standard run. We note that accurate mass will be used as a primary search variable while the retention time of the glycan species is to be used as a confirmatory variable in the glycan screening method. The retention time information needs to be updated for each separation gradient used for the glycan analysis.

### 3.3 Comparison of VRC01 Glycosylation From Four Different Clones

Several clonal CHO cell lines expressing VRC01 mAb were evaluated for the following criterion: the clone should have maximum viable cell densities ranging between 1.0–2.0 × 10^7^ cells/ml and enough volumetric productivity over a standard 14 days fed-batch platform process to ensure that enough material for analysis of N-linked glycans was generated for analysis. Further, the glycan profile should be within a range that allows for any change to be reliably measured in every aspect of the glycosylation profile: galactosylation, fucosylation, sialylation, and high mannose content.

We conducted eight independent fed-batch experiments using the glycan screening method to select one out of the four available clones (two replicates for each clone) for further evaluation. Each of the clones exhibited different volumetric productivities and were developed using a DHFR-based MTX selection system and the detail of their development is provided in a recent publication (Nmagu et al., 2021). The clones, specifically A11, CP24, CP19, and CP 35 demonstrated titers of 1,770, 1,550, 750, and 61 mg/L (Nmagu et al., 2021).

**FIGURE 3:**
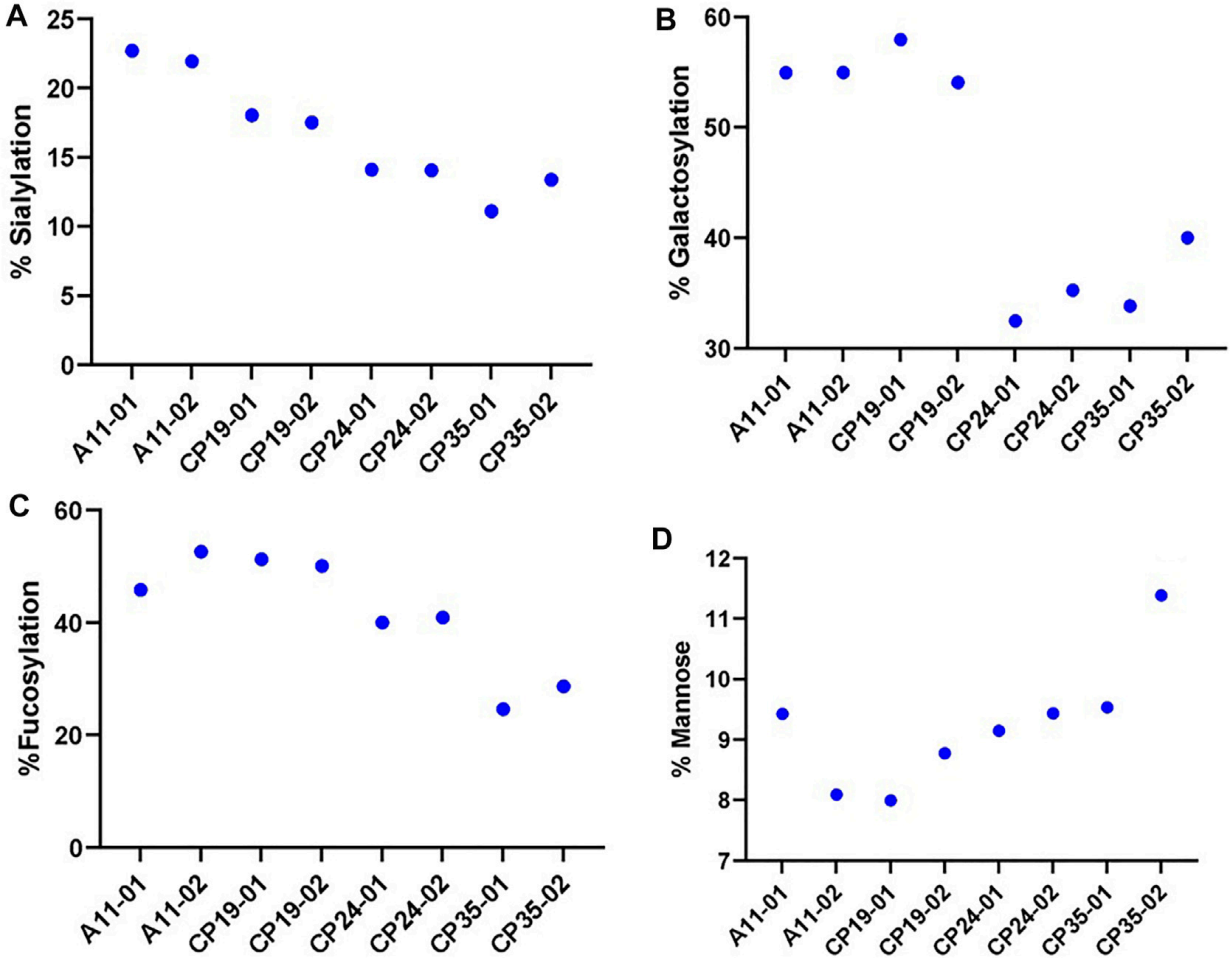
The relative levels of various glycan attribute. **(A)** Sialylation, **(B)** Galactosylation, **(C)** Fucosylation and **(D)** Mannose content for four different CHO clones (A11, CP19, CP24, and CP35) expressing VRC01 mAb in a 12 days long fed-culture. The suffix 01 and 02 represents the biological replicates for each of the clone.

The glycosylation profiles of VRC01 from these clones show large reproducible differences in the levels of sialylation, galactosylation, and fucosylation. As shown in Figure 3, the highest producing clone A11, exhibited maximum relative abundance of the levels of sialylation (∼24%), galactosylation (55%), and fucosylation (50%). On the other hand, the lowest producing clone CP35 exhibited the lowest relative abundance of sialylation (10%), galactosylation (35%), and fucosylation (25%). The intermediate producer clones, CP19 and CP24, resulted in relative levels of sialylation, and fucosylation that were less than A11, but higher than CP35. The galactosylation levels in CP24 and CP35 clones were 33.9 ± 1.97 and 36.9 ± 4.36, thereby indicating that the changes are not statistically significant between these 2 cell lines. However, it was found that while the productivity of CP24 cell lines was more than CP19, the levels of the sialylation, galactosylation, and fucosylation were higher in CP19 indicating that the glycosylation levels does not follow a linear relationship with the productivity of the clone. All four clones exhibited different glycan profiles indicating variability in the glycan processing machinery potentially attributed to differential availability of nucleotide sugars, glycan precursors, conformational steric hindrance, and metabolic activity of each clone.

There was no significant difference in the levels of the high mannose glycoforms (M5 forms) in VRC01 from any of the four clones indicating that the structural diversification from the immature high mannose N-glycans into relatively more processed and functionally mature forms consisting of hybrid and complex N- glycans subtypes did take place, regardless of the differences in the productivity of the clones. All clones exhibited similar viable cell density and viability profiles through day 3 before diverging, likely as a result of innate clonal variations. Clone CP35 seemed to diverge the most as it exhibited the highest VCD with nearly 50% greater cell density during exponential growth. This increase in exponential growth for CP35, correlated with greater glucose/nutrient consumption and lactate production which may be associated with apoptosis, leading to a shortened fed-batch culture duration in comparison to the other cell clones. Meanwhile, clone A11 showed slightly greater late culture viability than CP19 and CP24, and A11 had a distinctively lower lactate profile than the other cell clones. Based on these characteristics, the A11 clone was selected for further studies to explore glycosylation dynamics of VRC01 under different culture conditions.

### 3.4 Day-Wise Evolution of VRC01 Glycans in a Fed-Batch CHO Cell Culture

The A11 CHO clone was cultivated in a 14 days fed-batch cell culture process, carried out in a 25 ml shake flask. HILIC was used for VRC01 glycan analysis from cell-culture harvest samples collected on different days across exponential growth (days 5, 7, and 8) and the stationary production phase (days 9 and 11) (Nmagu et al., 2021). The individual glycan structures were then grouped into major glycosylated species, namely high mannose, galactosylated, sialylated, fucosylated, and afucosylated species.

**FIGURE 4:**
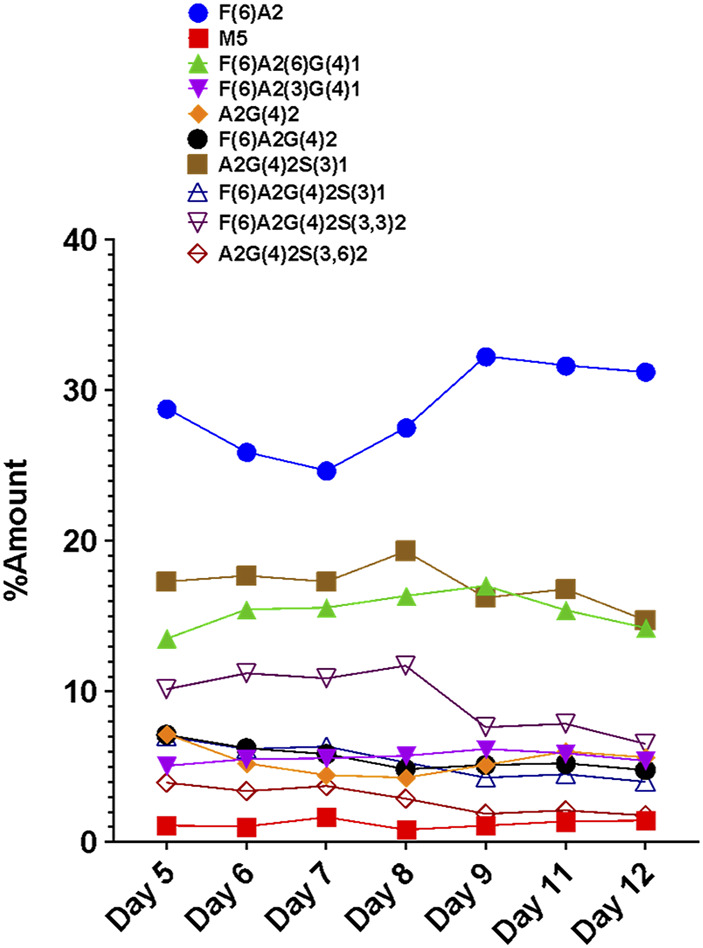
Day-wise evolution of various glycan species of the VRC01 mAb expressed using A11 CHO cell line in fed-batch culture.

The FLR traces of the monitored glycans of the VRC01 for the respective days are shown in Figure 4. On day 5, all 12 monitored glycans species were detected and identified, though some were low abundance. With the increasing cell culture duration until day 8, the relative abundance of certain glycan species such as F(6)A2, F(6)A2(6)G(4)1, F(6)A2(3)G(4)1, increased. However, the amounts of F(6)A2G(4)2, F(6)A2G(4)2S(3)1, F(6)A2G(4)2S(3,3)2, A2G(4)2S(3)1 remained relatively constant from day 5 to day 8 (growth phase). From day 8 onwards, the relative abundances of the latter glycan species started decreasing, while the former glycans remained unchanged. The relative abundance of the M5 species remained the same throughout the cell culture period. These data are consistent with a bottleneck in the VRC01 glycosylation that is not in the ability to convert high mannose into low mannose (and followed by the subsequent extension of the glycosylation network in the Golgi), but with steps leading to the transfer of galactosyl and sialyl transfer might be the rate-limiting step in overall glycosylation reaction of the VRC01 in CHO cells. The relatively constant levels of glycoforms such as A2G(4)2S(3)1, F(6) A2G(4)2S(3)1, F(6)A2G(4)2S(3,3)2, and A2G(4)2S(3,6)2 (all of them representing terminally sialylated forms) was observed during the cell culture phase when the viable cell density (VCD) was increasing. However, as the VCD started to decline from day 8 onwards, the relative abundances of the sialylated glycoforms also started decreasing. This decrease in the levels of sialylated glycans at later days of the cell culture (beyond day 8) might be attributed either to the availability of necessary nucleotide sugar donors (NSDs) or the expression levels of the enzymes responsible for the transfer of sialic acid to the growing glycosylation network on the VRC01, or both.

### 3.5 Comparison of VRC01 Glycosylation Grown in Three Different Media

**FIGURE 5:**
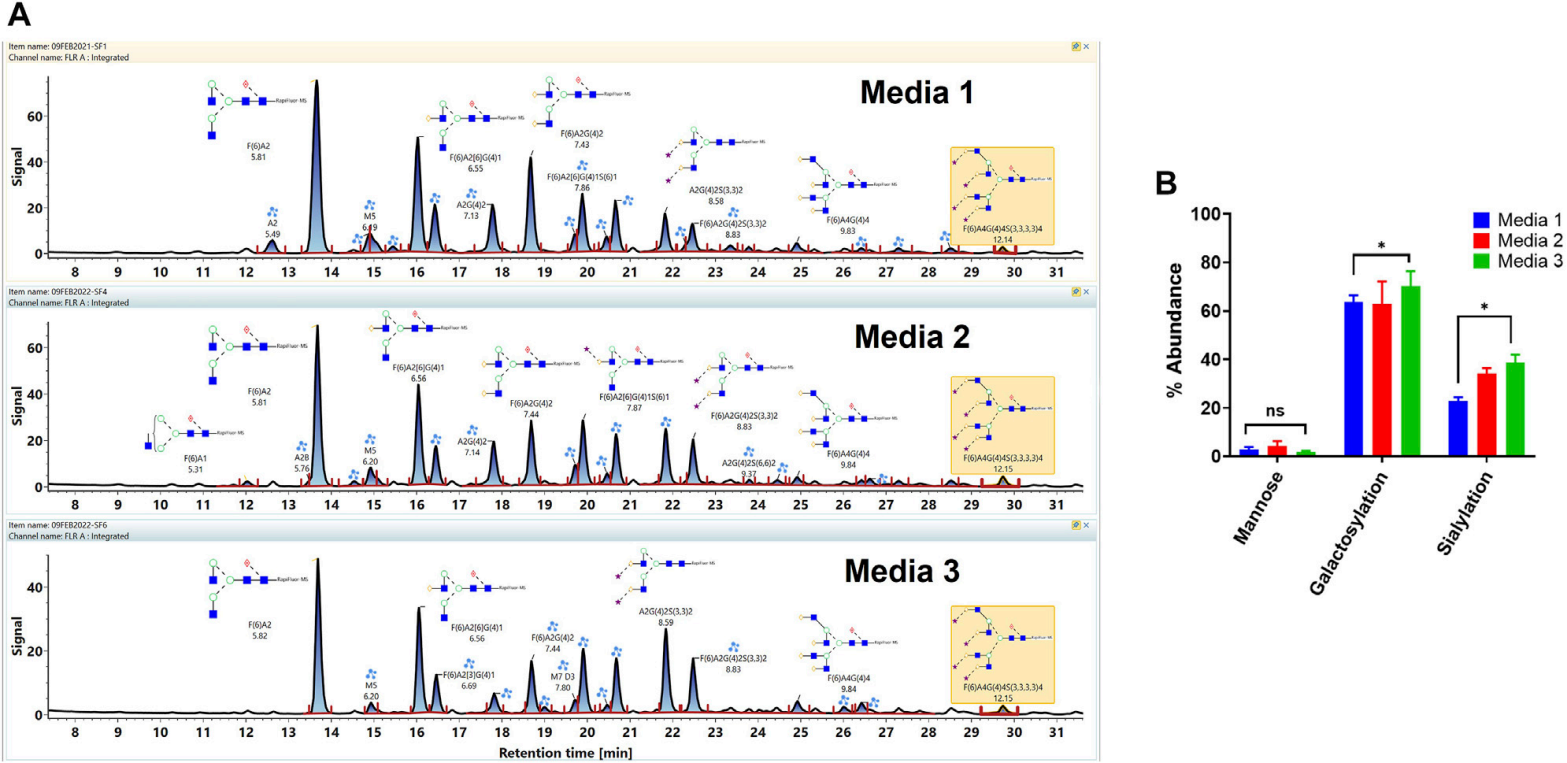
**(A)** Released glycan map (FLR trace) of the VRC01 expressed using A11 clone in three different media. **(B)** Relative abundance of various glycan attributes of VRC01 grown in the three different media.

The workflow was used for the assessment of the impact of the different cell culture media on the VRC01 glycosylation characteristics. Here, the VRC01 mAb were expressed in a fed-batch CHO culture in both bioreactor and the shake-flask modes of operation in three different media (Media 1, 2, and 3). As shown in Figure 5A, glycans of the VRC01 grown in Media 1 and 2 show similar relative abundances. In contrast, we observed a lower abundance of all glycan species from samples grown in Medium 3 as evidenced by the signal intensity in Figure 5A. The Man 5 species was ∼27 and ∼52% less in Media 2 and 3, respectively, as compared to that of Medium 1. Similarly, the afucosylated content (mainly from the A2G(4)2 and A2G(4)2S(3,3)2 species) constituted 11.2, 14.9, and 15.6% respectively in Media 1, 2, and 3. Another significant difference in the VRC01 glycosylation that was seen across the three media was in levels of sialylation which exhibited the following trend: sialylation (Medium 3)> sialylation (Medium 2) > sialylation (Medium 1). The increase in sialylation in Media 2 and 3 were attributed to the increase in the relative abundances of following glycan species in Media 2 and 3, respectively as compared that in Medium 1: F(6)A2[6]G(4)1S(6)1 (25.6 and 29.9%), F(6)A2G(4)2S(3)1 (17 and 28.2%), A2G(4)2S(3,3)2 (39.6 and 62%), F(6)A2G(4)2S(3,3)2 (49.2 and 59.3%), F(6)A3G(4)3S(3,3,3)3 (15.3 and 50%), and F(6)A4G(4)4S(3,3,3,3)4 (57 and 56%). Thus, we generally observed a modest increase in the levels of mono-sialylation, and a significant increase in the levels of di-, tri-, and tetra-sialylation of VRC01 glycans in Media 2 and 3, respectively as compared to Medium 1. In addition, consistent with this trend, a modest increase of 12.7 and 21.2% were noted in the levels of galactosylation of VRC01 grown in Media 2 and 3 as compared to Medium 1 (Figure 5B).

The low levels of high mannose glycans along with higher levels of afucosylated glycans seen in Media 2 and 3 as compared to Medium 1 suggests a positive impact of cell culture medium composition on the product quality attribute of VRC01 in the former as compared to the latter. The presence of high mannose glycans in biotherapeutics has been correlated with their rapid clearance in the body resulting in decreased half-life of the molecule. Similarly, a positive correlation between the levels of afucosylated glycans and the antibody-mediated cell cytotoxicity has been established. Recently, a positive correlation in the levels of sialylation and anti-inflammatory activity has also been observed for several mAbs.

### 3.6 Comparison of VRC01 Glycosylation Grown Under Two Different Process Conditions

Finally, the glycan profile of VRC01 grown in the Ambr250 bioreactor under two different process conditions was assessed using a glycan screening workflow. The two processes differed from each other in terms of the feedback loop used for DO control over the course of the cell culture (Supplementary Figure S1). The processes are referred to as 2-level DO and 6-level DO, respectively.

**FIGURE 6:**
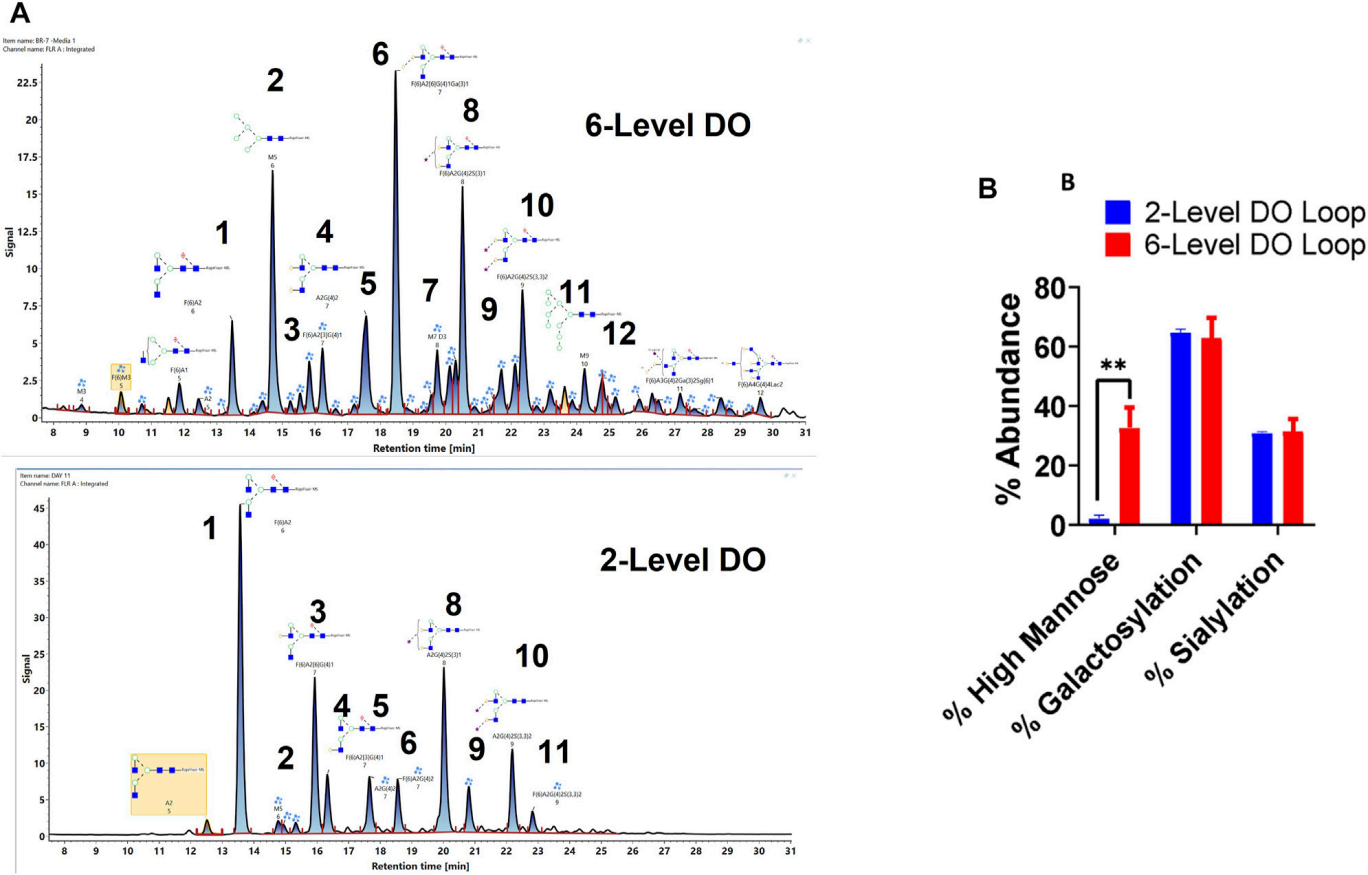
**(A)** Released glycan map (FLR trace) of the VRC01 expressed using A11 clone using two different processes in Ambr 250 bioreactor. **(B)** Relative abundance of various glycan attributes of VRC01 grown under the two process conditions.

As seen in Figure 6, there are substantial differences in the glycosylation profiles of the VRC01 expressed under these two conditions. The 6-level DO process resulted in the presence of greater than ∼10-fold higher mannose forms in the VRC01 than the 2-level DO process. This difference is evident from the presence of M5, M7, and M9 forms (peaks 2, 7, 12 in Figure 6A) in the former and was completely absent (except trace levels of M5) in the latter. Even though the overall galactosylation levels of the VRC01 from both processes were similar, several compositional differences of individual glycan forms were also noted between the two processes. For example, while the ratio of peak areas of G1F isoforms (peaks 3 and 4) is 3:1 for the 2-level DO process, it is almost 1:1 for the 6-level DO process. However, the G2F species (peak 6) is significantly higher in abundance in the 6-level DO process than the 2-level DO process, thereby compensating for the decrease in the G1F form. There was no significant change in the sialylated forms (peaks 8, 9,10, and 11), which resulted in a similar level of overall sialylation in the VRC01 from both processes.

Thus, the workflow can highlight the impact of process changes on the observed glycan heterogeneity of mAbs from CHO cell cultures.

## 4 Discussion

A major consideration with the CHO-based mAb production is the need to assess the glycan heterogeneity. Several factors, including process parameters, media and feed compositions, cell culture duration, and feeding strategy impacts glycan heterogeneity (Batra and Rathore, 2016). It is important to understand the source of glycan heterogeneity during mAb production to devise strategies to minimize the glycosylation variations observed during manufacturing. Towards this goal, an analytical method that can provide a comprehensive mAb glycosylation profile by separating and resolving as many glycan species as possible is of significant interest. The current approach of performing such an analysis is by the enzymatic release of the glycans from the mAbs followed by their separation and detection using LC-FLR-MS (Hilliard et al., 2017).

In the present study, a screening method for glycan analysis is developed that leverages the accurate mass and retention time of the glycan species for their identification and annotation. The proposed method has an advantage over the existing released glycan workflows regarding its ability to optimize the separation gradient that can help improve the resolving power of the chromatography. Additionally, the method does not require calibration using the dextran ladder for each analysis campaign as only the accurate mass and retention time is used for identification and annotation of the glycan species in the released glycan map, thus the method offers advantages as a screening approach as a surrogate to other methods that may be validated.

The method was applied to understand the glycan heterogeneity of VRC01 under various processing conditions. The existing literature attributes cell culture duration as one of the most dominant factors that bring about changes in the N-glycan distribution, gene expression profiles, metabolic states, and changes in the cell cycle (Bibila and Robinson, 1995; Hossler et al., 2009; Pacis et al., 2011). A general trend that is observed for different host cell lines, mAbs, and feeding strategies is an increase in the specific productivity with a simultaneous decrease in the levels of galactosylation over the course of typical fed-batch cell culture (Madabhushi et al., 2021). The decrease in the levels of galactosylation is more pronounced towards the end of the culture (Sumit et al., 2019). Our observations are consistent with this trend. The enhanced protein secretion capacity (at higher specific productivity) potentially corresponds to shorter Golgi residence time, but at the same time limits the glycosylation reactions leading to incomplete processing of the glycosylation resulting in lower galactosylation levels (Hossler et al., 2007; Kantardjieff et al., 2010; Val et al., 2016). However, our data on the glycosylation profiles of the VRC01 mAb expressed in CHO cell lines with a wide range of cell-specific productivities shows that the least productive clone also had the lowest galactosylation levels. This observation suggests that there are additional molecular mechanisms that may contribute.

Endogenous human IgG1 contains <1% of high mannose glycans (Mastrangeli et al., 2020). In contrast, recombinant therapeutic mAbs include ∼10% high mannose glycans (Goetze et al., 2011). Thus, there is interest in limiting excessive high mannose glycan formation in IgGs during commercial manufacturing (Mastrangeli et al., 2020). Current trends in biopharmaceutical manufacturing such as process intensification, development of fully humanized mAbs, growth in biosimilar products, development of high concentration mAb formulations, and the emergence of next-generation biopharmaceuticals such as anti-drug conjugates and bispecific antibodies, necessitates the control of high mannose content as a critical aspect in the process development (Berkel et al., 2009; Shi and Goudar, 2014; Feinberg et al., 2017; Sumit et al., 2019; Talbot et al., 2019; Mastrangeli et al., 2020).

Our analysis of the range of process conditions, cell lines, cell culture duration, and feed conditions indicate that high mannose glycans in VRC01 are unaffected by clone-specific productivity differences, standard commercial medium feeds (unless specific additives are used that are known to influence the high mannose glycan formation) and even the cell culture duration. However, under different DO control conditions in an AMBR250 bioreactor, it was found that there was a 10-fold increase in the high mannose content. The most abundant high-mannose structure observed was the M5 form, along with M3, M4, M6, M7, and M9 forms that were present in relatively very low abundance as compared to the M5 form. The occurrence of Man 5 glycans could potentially be attributed either by the action of mannosidases in the Golgi complex or from an incomplete/truncated biosynthesis of lipid linked oligosaccharides (LLO) in the endoplasmic reticulum (ER) (Breitling and Aebi, 2013; Mastrangeli et al., 2020). The M6, M7 glycans also could be an outcome of inefficient glycan processing in the Golgi complex, although they could also originate from incomplete LLO biosynthesis. Inefficient ER processing, potentially originating from a bottleneck in the availability of nucleotide sugar substrates such as UDP-GlcNAc and UDP-Man in the ER, thereby creating an imbalance in the protein folding and synthesis rates (Jiménez del Val et al., 2013; Mastrangeli et al., 2020) could also be a causative factor leading to the accumulation of high mannose glycans. Other process conditions that have been reported to cause ER stress during CHO cell cultures include a combination of factors such as temperature, pH, trace metals (zinc, copper), specific media supplements (Choline, spermine, ornithine) (Kang et al., 2015; Sou et al., 2015; Kuwae et al., 2018; Graham et al., 2019). In addition, there are reports in the literature showing that the extended cell culture durations also lead to less processing of glycans resulting in high mannose glycans (Jiang et al., 2018). Similarly, the impact of cell-specific productivity on the high mannose content is generally attributed to steric hinderance effects in the enzymatic processes, making the impact product-specific and less generalizable (Hills et al., 2001).

Another important mAb glycan attribute is the level of fucosylation. The high levels of fucosylation impose steric hindrance around the glycosylation site and thus inhibit the interaction of the Fc domain of the mAb with the Fc receptors on the effector cells leading to low antibody-dependent cell-mediated cytotoxicity (ADCC) (Pereira et al., 2018). However, existing data on commercial mAbs indicate only <10% of the recombinant mAbs as being non-fucosylated (Yamane-Ohnuki and Satoh, 2009). The most common strategies employed to reduce mAb fucosylation includes the use of engineered cell lines lacking the enzyme (α-1,6 fructosyltransferase) or overexpressing β(1-4)-N-acetylglucosaminyltransferase III gene (GnTIII), use of glycoside inhibitor kifunensine, and zinc finer mediated knockdown of fucose transport gene (Mimura et al., 2018). mAb fucosylation can also be varied by controlling the cell culture osmolarity and pH, where an inverse correlation exists (Konno et al., 2012). Here, we observed that the less-productive clone (CP35) also had the lowest fucosylation levels—consistent with observations from others. Also, the fucosylation level was observed to decrease with the loss in the cell viability at later days of cell culture (potentially due to accumulation of the lactate and ammonia that impacts the cell culture pH). The three different media used in the cell culture did not impact levels of the VRC01 fucosylation.

Together, our results illustrate that clonal variability, cell culture duration, media and feed, and process conditions together impact VRC01 mAb glycan heterogeneity. The reasons for glycan heterogeneity are multi-factorial and strategies to obtain a particular level of a particular glycoform towards achieving the desired product quality requires knowledge and adjustment of these parameters. The analytical method reported in this work provides a step towards elucidating and determining the complex relationship between process variables and glycosylation. Additional studies expanding on the data obtained from this study using multi-omic approaches will provide additional insights into the sources of glycan heterogeneity of mAbs during CHO cell culture and thereby aid in robust process development to control the glycosylation attributes to the desired levels.

## 5 Conclusion

The present study reports the development of a HILIC based glycan screening method for characterization of mAb glycosylation**.** The method employs the accurate mass of the glycans as the primary search variable and retention time as the confirmatory variable. The method was employed to study the source of glycan heterogeneity in a model IgG1 mAb, VRC01. It was determined that clonal heterogeneity, cell culture duration, and the process conditions impact several glycan attributes such as galactosylation, sialylation, fucosylation, and high mannose forms. Overall, the analytical method and glycosylation data presented here can guide in-depth analysis and process development to optimize the levels of VRC01 glycosylation for the desired product quality.

## Data Availability

The original contributions presented in the study are included in the article/Supplementary Material, further inquiries can be directed to the corresponding author.
