# Supplementary material for: Characterization of Monoclonal Antibody Glycan Heterogeneity Using Hydrophilic Interaction Liquid Chromatography-Mass Spectrometry
Source: Front Bioeng Biotechnol. 2022 Jan 11;9:805788. doi: 10.3389/fbioe.2021.805788 (PMC8786911; doi:10.3389/fbioe.2021.805788)
Supplement: Supplementary file 1 [file DataSheet1.PDF]

# Characterization of VRC01 monoclonal antibody glycan heterogeneity using hydrophilic interaction liquid chromatography-mass spectrometry

## Supplementary Figures

**Supplementary Table 1:** Listing the components of glycan screening library with their accurate mass information

| Name | Formula | Monoisotopic mass (g/mol) |
|---|---|---|
| M3 | C34H58N2O26 | 910.3278 |
| F(6)M3 | C40H68N2O30 | 1056.3857 |
| M4 | C40H68N2O31 | 1072.3806 |
| A1 | C42H71N3O31 | 1113.4072 |
| F(6)M4 | C46H78N2O35 | 1218.4385 |
| M5 | C46H78N2O36 | 1234.4334 |
| F(6)M3 GlcNAc1 | C48H81N3O35 | 1259.4651 |
| F(6)A1 | C48H81N3O35 | 1259.4651 |
| A1G(4)1 | C48H81N3O36 | 1275.46 |
| M4A1 | C48H81N3O36 | 1275.46 |
| A1[3]G(4)1 | C48H81N3O36 | 1275.46 |
| A2 | C50H84N4O36 | 1316.4865 |
| F(6)M5 | C52H88N2O40 | 1380.4913 |
| F(6)M5 | C52H88N2O40 | 1380.4913 |
| M6 D1 | C52H88N2O41 | 1396.4863 |
| M6 D2 | C52H88N2O41 | 1396.4863 |
| M6 D3 | C52H88N2O41 | 1396.4863 |
| M6 | C52H88N2O41 | 1396.4863 |
| F(6)A1[6]G(4)1 | C54H91N3O40 | 1421.5179 |
| F(6)M4A1 | C54H91N3O40 | 1421.5179 |
| F(6)A1G(4)1 | C54H91N3O40 | 1421.5179 |
| F(6)A1[3]G(4)1 | C54H91N3O40 | 1421.5179 |
| A1G(4)1Ga(3)1 | C54H91N3O41 | 1437.5128 |
| M5A1 | C54H91N3O41 | 1437.5128 |
| M5A1 | C54H91N3O41 | 1437.5128 |
| F(6)A2 | C56H94N4O40 | 1462.5444 |
| A2[3]G(4)1 | C56H94N4O41 | 1478.5393 |
| A2[6]G(4)1 | C56H94N4O41 | 1478.5393 |
| A2G(4)1 | C56H94N4O41 | 1478.5393 |
| A2B | C58H97N5O41 | 1519.5659 |
| A3 | C58H97N5O41 | 1519.5659 |
| M7 | C58H98N2O46 | 1558.5391 |
| M7 D3 | C58H98N2O46 | 1558.5391 |
| M7 D1 | C58H98N2O46 | 1558.5391 |
| A1G(4)1S(3)1 | C59H98N4O44 | 1566.5554 |
| F(6)M4A1G(4)1 | C60H101N3O45 | 1583.5707 |
| F(6)M5A1 | C60H101N3O45 | 1583.5707 |
| F(6)A1G(4)1Ga1 | C60H101N3O45 | 1583.5707 |
| M5A1G(4)1 | C60H101N3O46 | 1599.5656 |

| | | |
|---|---|---|
| M4A1G(4)1Ga(3)1 | C60H101N3O46 | 1599.5656 |
| F(6)A2[6]G(4)1 | C62H104N4O45 | 1624.5973 |
| F(6)A2[3]G(4)1 | C62H104N4O45 | 1624.5973 |
| A2G(4)2 | C62H104N4O46 | 1640.5922 |
| A2G(4)1Ga(3)1 | C62H104N4O46 | 1640.5922 |
| F(6)A3 | C64H107N5O45 | 1665.6238 |
| F(6)A2B | C64H107N5O45 | 1665.6238 |
| A2[6]BG(4)1 | C64H107N5O46 | 1681.6187 |
| M8 D1,D3 | C64H108N2O51 | 1720.5919 |
| M8 D2,D3 | C64H108N2O51 | 1720.5919 |
| M8 | C64H108N2O51 | 1720.5919 |
| A4 | C66H110N6O46 | 1722.6453 |
| F(6)A1G(4)1Sg(6)1 | C65H108N4O49 | 1728.6082 |
| F(6)M5A1G(4)1 | C66H111N3O50 | 1745.6235 |
| F(6)M4A1G(4)1Ga(3)1 | C66H111N3O50 | 1745.6235 |
| M5A1G(4)1Ga(3)1 | C66H111N3O51 | 1761.6184 |
| A2[6]G(4)1S(3)1 | C67H111N5O49 | 1769.6348 |
| A2[3]G(4)1S1 | C67H111N5O49 | 1769.6348 |
| A2[3]G(4)1S(3)1 | C67H111N5O49 | 1769.6348 |
| A2G(4)1Sg1 | C67H111N5O50 | 1785.6297 |
| F(6)A2[3]G1Ga1 | C68H114N4O50 | 1786.6501 |
| F(6)A2[6]G1Ga1 | C68H114N4O50 | 1786.6501 |
| F(6)A2G(4)1Ga(3)1 | C68H114N4O50 | 1786.6501 |
| F(6)A2G(4)2 | C68H114N4O50 | 1786.6501 |
| A2G(4)2Ga(3)1 | C68H114N4O51 | 1802.645 |
| F(6)A3G(4)1 | C70H117N5O50 | 1827.6766 |
| F(6)A2[3]BG(4)1 | C70H117N5O50 | 1827.6766 |
| F(6)A2[6]BG(4)1 | C70H117N5O50 | 1827.6766 |
| A2BG(4)2 | C70H117N5O51 | 1843.6715 |
| F(6)A3 GlcNAc1 | C72H120N6O50 | 1868.7032 |
| F(6)A4 | C72H120N6O50 | 1868.7032 |
| M9 | C70H118N2O56 | 1882.6447 |
| F(6)M4A1G(4)1Sg(6)1 | C71H118N4O54 | 1890.661 |
| M5A1G(4)1Sg(6)1 | C71H118N4O55 | 1906.656 |
| F(6)M5A1G(4)1Ga(3)1 | C72H121N3O55 | 1907.6764 |
| F(6)A2[6]G(4)1S(6)1 | C73H121N5O53 | 1915.6927 |
| F(6)A2[3]G(4)1S(3)1 | C73H121N5O53 | 1915.6927 |
| F(6)A2[6]G(4)1S(3)1 | C73H121N5O53 | 1915.6927 |
| F(6)A2[3]G(4)1S(6)1 | C73H121N5O53 | 1915.6927 |
| F(6)A2[6]G(4)1Sg(6)1 | C73H121N5O54 | 1931.6876 |
| F(6)A2[3]G(4)1Sg(6)1 | C73H121N5O54 | 1931.6876 |
| A2G(4)2S(3)1 | C73H121N5O54 | 1931.6876 |
| F(6)A2[6]G(4)1Sg(6)1 | C73H121N5O54 | 1931.6876 |
| A2G(4)2S(6)1 | C73H121N5O54 | 1931.6876 |
| F(6)A2G(4)2Ga(3)1 | C74H124N4O55 | 1948.7029 |
| A2G(4)2Ga(3)2 | C74H124N4O56 | 1964.6978 |

| | | |
|---|---|---|
| A2G2Ga2 | C74H124N4O56 | 1964.6978 |
| A2BG(4)1S(6)1 | C75H124N6O54 | 1972.7141 |
| F(6)A2BG(4)2 | C76H127N5O55 | 1989.7295 |
| F(6)A3G(4)2 | C76H127N5O55 | 1989.7295 |
| A3G(4)3 | C76H127N5O56 | 2005.7244 |
| A3G(4,4,3)3 | C76H127N5O56 | 2005.7244 |
| M10 a3D1,a3D3,a2D4(2) | C76H128N2O61 | 2044.6975 |
| F(6)M5A1G(4)1Sg(6)1 | C77H128N4O59 | 2052.7139 |
| F(6)A4 GlcNAc1 | C80H133N7O55 | 2071.7826 |
| F(6)A3GlcNAc2 | C80H133N7O55 | 2071.7826 |
| F(6)A2G(4)2S(3)1 | C79H131N5O58 | 2077.7455 |
| F(6)A2G(4)2S(6)1 | C79H131N5O58 | 2077.7455 |
| F(6)A2G(4)2Sg(6)1 | C79H131N5O59 | 2093.7404 |
| F(6)A2G(4)2Ga(3)2 | C80H134N4O60 | 2110.7557 |
| F(6)A2G2Ga2 | C80H134N4O60 | 2110.7557 |
| F(6)A2[6]BG(4)1S(6)1 | C81H134N6O58 | 2118.772 |
| A2BG(4)2S(6)1 | C81H134N6O59 | 2134.767 |
| F(6)A3G(4)3 | C82H137N5O60 | 2151.7823 |
| F(6)A3G(4)3 iso2 | C82H137N5O60 | 2151.7823 |
| M11 a3D1,[D2(1),D3(1)],a2D4(2) | C82H138N2O66 | 2206.7504 |
| A2G(4)2S(6,6)2 | C84H138N6O62 | 2222.783 |
| A2G(4)2S(3,3)2 | C84H138N6O62 | 2222.783 |
| A2G(4)2S(3,6)2 | C84H138N6O62 | 2222.783 |
| A2G(4)2Sg(3,6)2 | C84H138N6O64 | 2254.7728 |
| A2G(4)2Sg(6,6)2 | C84H138N6O64 | 2254.7728 |
| F(6)A2G(4)2Ga(3)1Sg(6)1 | C85H141N5O64 | 2255.7932 |
| F(6)A4 GlcNAc2 | C88H146N8O60 | 2274.8619 |
| F(6)A3 GlcNAc3 | C88H146N8O60 | 2274.8619 |
| F(6)A2BG(4)2S(6)1 | C87H144N6O63 | 2280.8249 |
| A3G(4)3S(6)1 | C87H144N6O64 | 2296.8198 |
| A3G(4)3S(3)1 | C87H144N6O64 | 2296.8198 |
| F(6)A3G(4)2Ga2 | C88H147N5O65 | 2313.8351 |
| M12 a3D1,[D2(2),D3(2)],a2D4(2) | C88H148N2O71 | 2368.8032 |
| F(6)A2G(4)2S(6,6)2 | C90H148N6O66 | 2368.8409 |
| F(6)A2G(4)2S(3,3)2 | C90H148N6O66 | 2368.8409 |
| A4G(4)4 | C90H150N6O66 | 2370.8566 |
| F(6)A2G(4)2Sg(3,6)2 | C90H148N6O68 | 2400.8307 |
| F(6)A2G(4)2Sg(6,6)2 | C90H148N6O68 | 2400.8307 |
| A2BG(4)2S(6,6)2 | C92H151N7O67 | 2425.8624 |
| F(6)A3G(4)3S(3)1 | C93H154N6O68 | 2442.8777 |
| F(6)A3G(4)3Sg(6)1 | C93H154N6O69 | 2458.8726 |
| F(6)A3G(4)3Ga2 | C94H157N5O70 | 2475.8879 |
| F(6)A3G(4)3Ga(3)2 | C94H157N5O70 | 2475.8879 |
| F(6)A4 GlcNAc3 | C96H159N9O65 | 2477.9413 |
| A3G(4)3Ga(3)3 | C94H157N5O71 | 2491.8828 |
| F(6)A3G(4)3Lac1 | C96H160N6O70 | 2516.9145 |

| | | |
|---|---|---|
| F(6)A4G(4)4 | C96H160N6O70 | 2516.9145 |
| M13 a3D1,[D2(3),D3(3)],a2D4(2) | C94H158N2O76 | 2530.856 |
| F(6)A2BG(4)2S(6,6)2 | C98H161N7O71 | 2571.9203 |
| A3G(4)3S(3,3)2 | C98H161N7O72 | 2587.9152 |
| A3G(4)3S(6,6)2 | C98H161N7O72 | 2587.9152 |
| A3G(4)3S(3,6)2 | C98H161N7O72 | 2587.9152 |
| F(6)A3G(4)2Ga(3)2Sg(6)1 | C99H164N6O74 | 2620.9254 |
| F(6)A3G(4)3Ga3 | C100H167N5O75 | 2637.9407 |
| F(6)A4 GlcNAc4 | C104H172N10O70 | 2681.0207 |
| M14 a3D1,[D2(4),D3(4)],a2D4(2) | C100H168N2O81 | 2692.9088 |
| F(6)A3G(4)3S(3,3)2 | C104H171N7O76 | 2733.9731 |
| F(6)A3G(4)3Ga(3)2Sg1 | C105H174N6O79 | 2782.9783 |
| F(6)A4G(4)4S(3)1 | C107H177N7O78 | 2808.0099 |
| A3S(6)1G(4,4,3)3S(3,3)2 | C109H178N8O80 | 2879.0106 |
| A3G(4)3S(3,3,6)3 | C109H178N8O80 | 2879.0106 |
| A3G(4)3S(3,6,6)3 | C109H178N8O80 | 2879.0106 |
| A3G(4)3S(6,6,6)3 | C109H178N8O80 | 2879.0106 |
| A3G(4)3S(3,3,3)3 | C109H178N8O80 | 2879.0106 |
| A3S(6)1G(4,4,3)3S(3,6)2 | C109H178N8O80 | 2879.0106 |
| F(6)A3G(4)3Lac2 | C110H183N7O80 | 2882.0467 |
| F(6)A4G(4)4Lac1 | C110H183N7O80 | 2882.0467 |
| A4G(4)4S(3,3)2 | C112H184N8O82 | 2953.0474 |
| F(6)A3G(4)3S(3,3,3)3 | C115H188N8O84 | 3025.0685 |
| F(6)A4G(4)4S(3,3)2 | C118H194N8O86 | 3099.1053 |
| A3S(6)1G(4,4,3)3S(3,6,6)3 | C120H195N9O88 | 3170.106 |
| A3S(6)1G(4,4,3)3S(6,3,3)3 | C120H195N9O88 | 3170.106 |
| A3S(6)1G(4,4,3)3S(3,3,3)3 | C120H195N9O88 | 3170.106 |
| A3S(6)1G(4,4,3)3S(6,3,3)3 | C120H195N9O88 | 3170.106 |
| F(6)A3G(4)3Lac3 | C124H206N8O90 | 3247.1789 |
| F(6)A4G(4)4Lac2 | C124H206N8O90 | 3247.1789 |
| F(6)A4G(4)4S(3,3,3)3 | C129H211N9O94 | 3390.2007 |
| F(6)A3G(4)3Lac1S3 | C129H211N9O94 | 3390.2007 |
| A4G(4)4S(3,3,3,3)4 | C134H218N10O98 | 3535.2382 |
| F(6)A4G(4)4Lac3 | C138H229N9O100 | 3612.3111 |
| FA4G(4,4,4,4)4S(3,3,3,3)4 | C140H228N10O102 | 3681.2961 |
| F(6)A4G(4)4Lac1S3 | C143H234N10O104 | 3755.3329 |
| F(6)A3G(4)3Lac2S3 | C143H234N10O104 | 3755.3329 |
| F(6)A4G(4)4Lac3S1 | C149H246N10O108 | 3903.4065 |
| F(6)A4G(4)4Lac4 | C152H252N10O110 | 3977.4433 |
| F(6)A4G(4)4Lac1S4 | C154H251N11O112 | 4046.4283 |
| F(6)A3G(4)3Lac3S3 | C157H257N11O114 | 4120.4651 |
| F(6)A4G(4)4Lac2S3 | C157H257N11O114 | 4120.4651 |
| F(6)A4G(4)4Lac3S2 | C160H263N11O116 | 4194.5019 |
| F(6)A4G(4)4Lac4S1 | C163H269N11O118 | 4268.5387 |
| F(6)A4G(4)4Lac2S4 | C168H274N12O122 | 4411.5605 |
| F(6)A4G(4)4Lac3S3 | C171H280N12O124 | 4485.5973 |

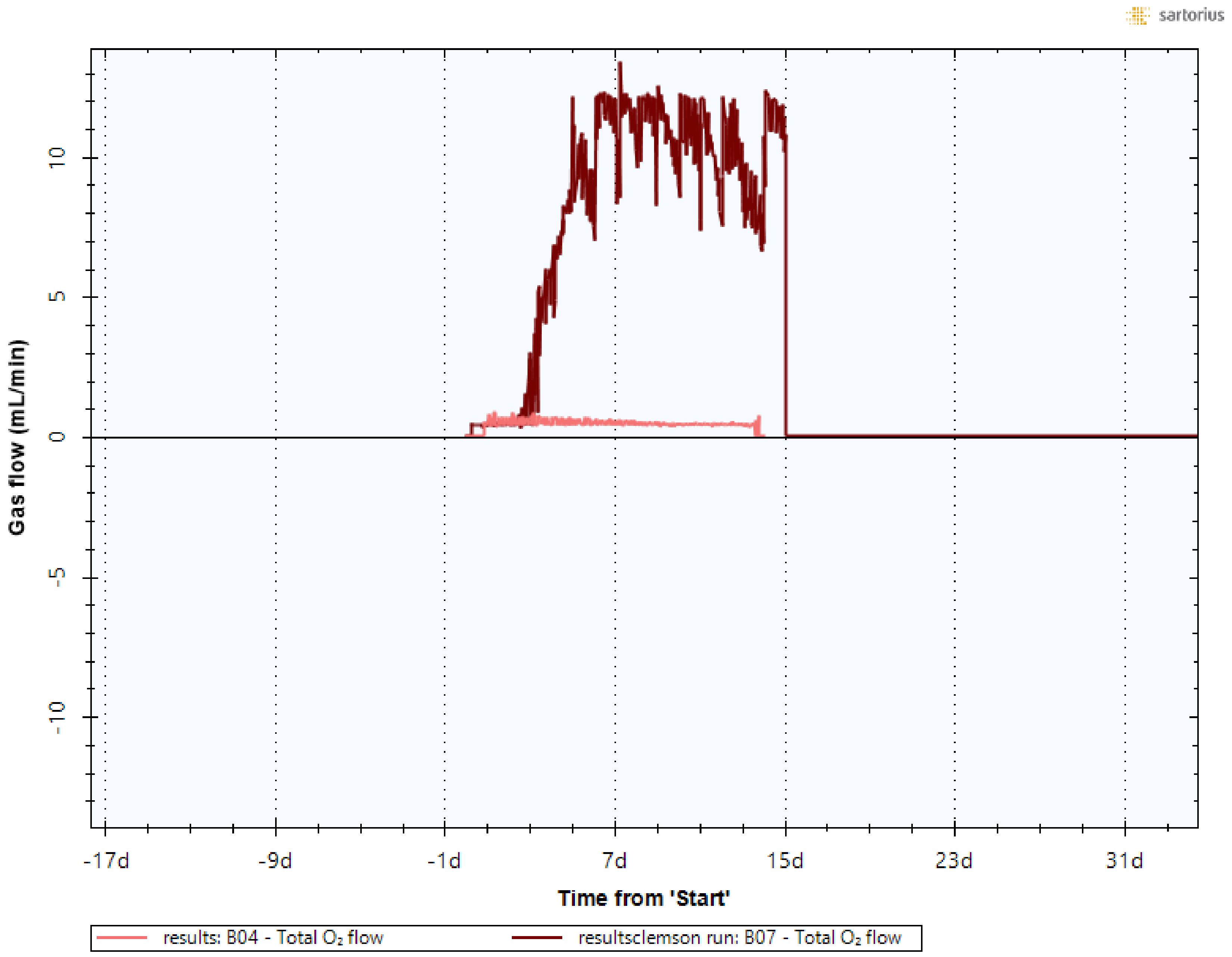


Supplementary Figure 1 : DO profiles in 2-level and 6-level Control system during CHO cell culture
